# Subnanometer Thick Native sp^2^ Carbon on Oxidized Diamond Surfaces

**DOI:** 10.1021/acs.langmuir.5c02616

**Published:** 2025-10-01

**Authors:** Ricardo Vidrio, Cesar Saucedo, Vincenzo Lordi, Shimon Kolkowitz, Keith G. Ray, Robert J. Hamers, Jennifer T. Choy

**Affiliations:** 1 Department of Electrical and Computer Engineering, 5228 University of Wisconsin-Madison, 1415 Engineering Dr, Madison, Wisconsin 53706, United States; 2 Department of Chemistry, University of Wisconsin-Madison, 1101 University Ave, Madison, Wisconsin 53706, United States; 3 4578 Lawrence Livermore National Laboratory, 7000 East Ave, Livermore, California 94550, United States; 4 Department of Physics, University of California, Berkeley Physics South Hall, Berkeley, California 94720-7300, United States; 5 Physical Electronics, 18725 Lake Dr E, Chanhassen, Minnesota 55317, United States

## Abstract

Oxygen-terminated diamond has a wide breadth of applications, which include stabilizing near-surface color centers, semiconductor devices, and biological sensors. Despite the vast literature on characterizing functionalization groups on diamond, the chemical composition of the shallowest portion of the surface (<1 nm) is challenging to probe with conventional techniques like XPS and FTIR. In this work, we demonstrate the use of angle-resolved XPS to probe the first ten nanometers of both oxygen and hydrogen terminated (100) single-crystalline diamond grown via chemical vapor deposition (CVD). With the use of consistent peak-fitting methods, the peak identities and relative peak binding energies were identified for sp^2^ carbon, ether, hydroxyl, carbonyl, and C–H groups for both of these diamond surface terminations. For the oxygen-terminated sample, we also quantified the thickness of the sp^2^ carbon layer situated on top of the bulk sp^3^ diamond bonded carbon to be 0.3 ± 0.1 nm, based on the analysis of the Auger electron spectra and D-parameter calculations. These results indicate that the majority of the oxygen is bonded to the sp^2^ carbon layer on the diamond, and not directly to the sp^3^ diamond bonded carbon.

## Graphical abstract

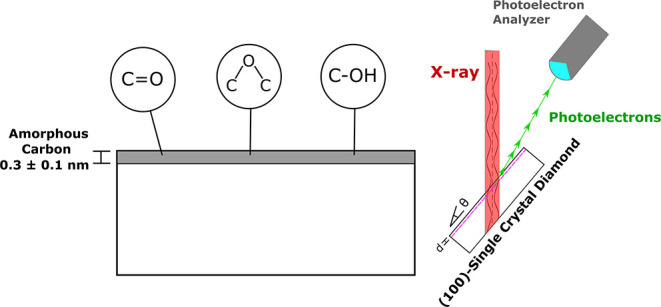

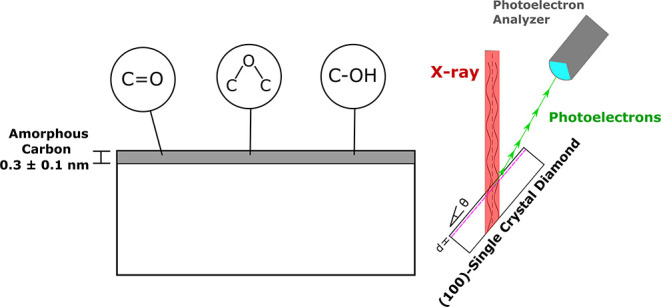

## Introduction

Depending on the type of surface termination employed, single-crystal diamonds (SCDs) can be tailored for a wide variety of applications ranging from DNA sensing,^1^ radiation detection,^2,3^ and stabilizing resonating nanostructures.^4^ For example, oxygen (O)-terminated SCD has been shown to be an effective Schottky barrier diode using selective growing procedures and careful nanofabrication methods,^5^ while hydrogen (H) -terminated SCD has shown promise towards becoming viable for the detection of deep-ultraviolet light.^6^ Quite recently, SCDs with either O or Nitrogen (N)-terminated diamond hold potential toward enhancing the spin and optical properties of near-surface color centers, thus enabling the use of SCD surfaces for the next generation of quantum engineering applications.^7–10^ And although there are many types of diamonds, such as nanocrystalline diamond, boron-doped diamonds, and diamond-like carbon films, here we focus on undoped SCD, which tends to be most prevalently used in quantum sensing^11^ and radiation detection.^3^

Though there is a plethora of literature available on the study of functionalized SCD surfaces,^12–15^ analysis of the shallowest portion of the surface proves difficult with common materials characterization techniques. While Fourier Transform Infrared (FTIR) spectroscopy is capable of discerning O functional groups on diamond materials,^16–20^ it is unfortunately not surface-sensitive enough, as the penetration depth is on the order of microns. Despite Raman spectroscopy being able to discern the sp^2^ and sp^3^ carbon (C) information on diamond,^21^ depth-profiling Raman studies are limited to analyzing depths on the order of microns or hundreds of nanometers, which are depths well within the order of bulk diamond and not diamond surfaces.^22,23^ Furthermore, diamond is one of the hardest materials on Earth,^24^ making milling or machining for other methods, such as scanning tunnel microscopy (STM) or atom probe tomography (APT), difficult, particularly so for SCD.

Although the prevailing model for the SCD surface has consisted of solely sp^3^ C bonds, which are directly bonded to surface functionalizations,^25–28^ recent experimental work, via angle-resolved X-ray photoelectron spectroscopy (ARXPS), has provided evidence for the existence of a superficial layer of sp^2^ C, which rests on the bulk sp^3^ diamond-bonded C^29^. Similarly, depth-profiling measurements on nitrogen-terminated polycrystalline diamond (PCD) samples have yielded conclusive evidence of the existence of graphene-like islands on the PCD surface.^30^ Furthermore, studies investigating O-termination treatments of SCD, such as wet chemistry, dry chemistry, O plasma methods, have consistently reported detectable levels of sp^2^ C in the C 1s XPS spectra.^8,12,29,31–34^ Given the importance of diamond research in quantum and semiconductor technologies, an encompassing model of the surface of diamond will be useful for devising effective surface preparation techniques and accurate analysis of device performance. This proves especially pertinent in the diamond quantum sensing community, as amounts of sp^2^ C have been found to be detrimental to spin coherence in near-surface NV centers, which in turn limits measurement sensitivity.^35^

In this work, we performed ARXPS on an O-terminated (100) diamond grown using chemical vapor deposition (CVD), which enabled us to identify the multilayered chemical structure in the first 10 nm below the diamond surface. We begin by interpreting the C 1s spectra of two different surface treatments of (100) single-crystalline CVD diamond, a H-terminated and an O-terminated sample, at multiple photoelectron emission angles. We compare these two different surfaces as both H- and O-terminated diamond are commonly used within the quantum and semiconductor community for a host of applications.^31,8,36,37^ Additionally, the comparison illustrates how both sp^2^ C and surface terminations are constrained to only the most superficial portions of the diamond. We show that the respective terminations on the diamond become less pronounced as the photoelectrons emanate from the deeper sp^3^ C. Furthermore, we also assign regions on the spectra with their corresponding chemical identities, as well as report relative binding energy peak values for the singly bonded C–O groups, carbonyls, sp^2^ C, and C–H peaks. Next, we analyze the C Auger electron lineshapes (CKLL) and calculate the D-parameter to determine the evolution from the superficial amorphous sp^2^ C layer to the bulk sp^3^ C diamond for the O-terminated sample. Given the considerably shorter in elastic mean free path for Auger electrons in diamond compared with core electrons, our technique probes a much shallower region of the diamond surface. This, combined with the stage-tilting capabilities from ARXPS, allows us to probe subnanometer depth information from the SCD sample, giving us an unprecedented amount of information about the molecular nature of the C responsible for the O-terminations. We conclude that the sp^2^ C layer comprises only the first 0.3 nm of the surface. While prior studies^35,38,32^ have reported on the presence of sp^2^ C on O-terminated diamond surfaces, this finding elucidates the bonding characteristic of this native sp^2^ C layer, namely that the O content is bonded to the sp^2^ C and not directly on the bulk sp^3^ C.

## Experimental Methods

### Sample Preparation

All diamond samples used here are type IIa (100) SCD grown using CVD purchased from Element 6, with a surface roughness specified by the manufacturer, polished to within <30 nm. Two different surface treatments were studied, the O-termination and the H-termination. The O-termination was accomplished by taking as-received diamond samples and performing a triacid clean bath, which consists of a 1:1:1 volumetric mixture of perchloric, sulfuric, and nitric acid at a constant temperature of 450 °C, which results in a diamond surface of at least 4.8% O 1s atomic percentage.^32^ The H-terminated sample was first triacid cleaned and then put in a hydrogen plasma chamber to induce an effective H-termination. Diamond samples were placed in a quartz vacuum chamber, which was subsequently evacuated to pressures of ∼25 mTorr. A hydrogen plasma was then created at a microwave power of 600 W plasma for 15 min, followed by cooling under constant hydrogen flow (100 sccm, ∼3 Torr) for 1 h.

We confirm through atomic force microscopy (AFM) measurements that the O-terminated and H-terminated samples have surface roughness below 1 nm, with representative AFM images shown in the Supporting Information (Section S.1).

### ARXPS Measurements

A PHI VersaProbe III with monochromatic X-rays from an aluminum anode was utilized, and multiple scans were collected, including a survey scan, a narrow scan of the C 1s spectra, a narrow scan of the O 1s spectra, and a narrow scan of the CKLL. Survey scans were performed with a pass energy of 280 eV and a step size of 1 eV. Narrow scans of the C 1s and O 1s were performed with a pass energy of 26 eV and a step size of 0.1 eV. The flood gun was used at all times during the measurements to mitigate the effects of peak shifting. The PHI VersaProbe IIII comes equipped with stage-tilting capabilities that change the angle at which the photoelectrons are emitted from the sample, allowing for the study of the photoelectron spectra at different depths.

Shown in Figure 1a is a schematic that illustrates the angle-tilting capabilities in the PHI VersaProbe III. The angle-tilting in this experiment progressed from a minimum angle of 10°, which represents the photoelectrons coming from the shallowest portions of the diamond, toward a maximum angle of 90°, in which the ejected photoelectrons come in from the bulk of the diamond. All angles come with an experimental uncertainty of 6°, limited by the omission of the aperture in the ARXPS setup in order to maximize signal-to-noise ratio (SNR) in the photoelectron spectra. This angle uncertainty represents the uncertainty in the collection angle for the photoelectron analyzer.

**Figure 1:**
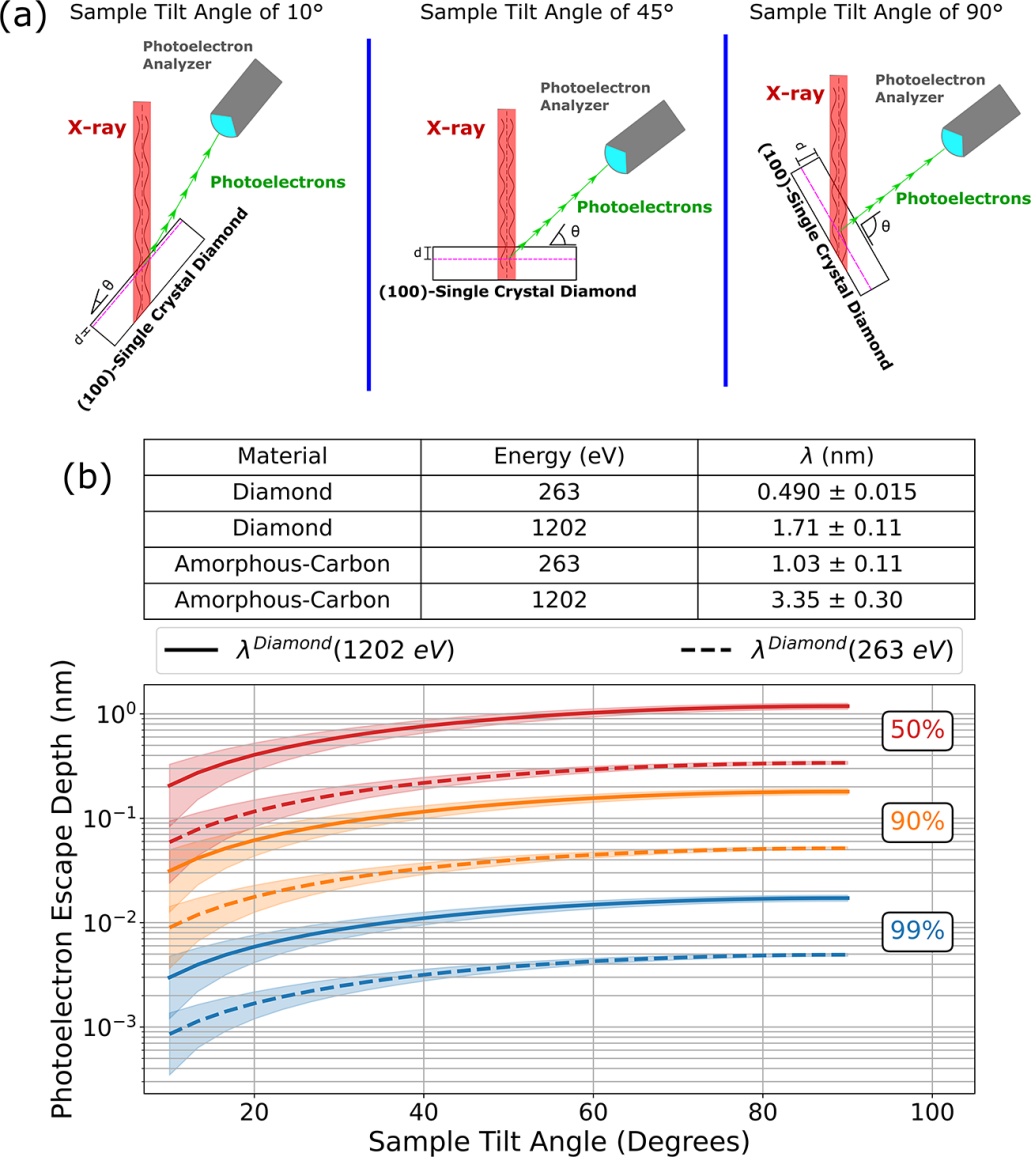
(a) Experimental setup of the diamond sample within the PHI VersaProbe III. (b) Photoelectron escape depth as a function of sample tilt angle shown at three different escape probabilities: 50, 90, and 99%. Shaded regions on the figure represent the uncertainty in the photoelectron escape depth given uncertainties in IMFP and acceptance angle.

### Evaluation of the Surface Sensitivity of Photoelectron Spectra

To estimate the escape depths of the photoelectrons from the diamond sample, the photoelectron escape probability equation was used (eq 1).

$$P(z,\theta )=e^{-z/\left(\lambda \cdot \sin(\theta )\right)}$$ 1

Here, *z* represents the depth of the diamond at which the photoelectron originates, in nm; λ represents the inelastic mean free path (IMFP) of the photoelectron; and θ is the sample tilt angle relative to the detector. The value of λ depends on the photoelectron kinetic energy and sample material, which is taken to be either diamond or amorphous carbon. For this work, the relevant photoelectron energies are at 1202 eV (for core C 1s electrons) and 263 eV (for Auger electrons); the corresponding λ values were obtained from the literature,^39,40^ along with their corresponding experimental uncertainties, and are shown in Figure 1b (top panel). Photoelectron refraction effects were also considered in the interpretation of photoelectron emission data (Supporting Information S.2) but were determined to contribute to an angle uncertainty smaller than 1° and thus can be neglected in the analysis.

We can evaluate the photoelectron escape depth for a given escape probability (say 50, 90, and 99%), plotted as a function of sample tilt angle. Figure 1b highlights the surface sensitivity of the XPS method, as both the C 1s and CKLL electrons are 50% likely to originate from roughly the first 1.2 nm of the surface throughout all angles surveyed during the experiment. Furthermore, throughout all escape probabilities, we can observe that there is a mismatch between the depths that are sampled between the C 1s and CKLL spectra, as the CKLL spectra are probing shallower depths when compared to the C 1s spectra due to the differing λ values. This means that the CKLL electrons are roughly three times as surface sensitive as their C 1s counterparts (see eq 1 and Figure 1).

### Analysis of XPS Spectra

All C 1s XPS peak fitting and atomic quantification were performed on CasaXPS software.^41^ A tougaard background was utilized for all peak fits, with the full width half-maximum being constrained to within 0.6 to 0.9 eV for the sp^3^ C peak and 1.3 to 1.6 eV for the sp^2^ C, singly bonded oxygen carbon groups C–O, and the doubly bonded oxygen carbon groups C=O. The C–O peak corresponds to the oxygen functional groups comprising hydroxyl and ether groups,^42,43^ whereas the C=O peak is comprised of carbonyl bonds.^8^ Analysis of the CKLL spectra was performed on Python with the use of a modified form of the Whittaker filter, known as the Whittaker–Eilers smoothening method.^44,45^

## Results and Discussion

### Comparison of H-Terminated and O-Terminated Surfaces

We first compared the C 1s spectra for the H- and O-terminated diamond at different sample tilt angles (Figure 2). The arrows point to approximate peak regions corresponding to the unique chemical species which have been identified in prior works^8,32,46,33,47^ and have been successfully deconvoluted using multiparameter peak-fitting methods^32,48–52^ described in Analysis of XPS Spectra. A representative plot that illustrates the results of one of the peak fits for the C 1s spectra at a sample tilt angle of 10° is shown in Figure 3. A similar process of peak fitting was performed across all C 1s spectra for all eight sample tilt angles. The results of three additional peak fits are presented in the Supporting Information (Section S.3) of this work.

**Figure 2:**
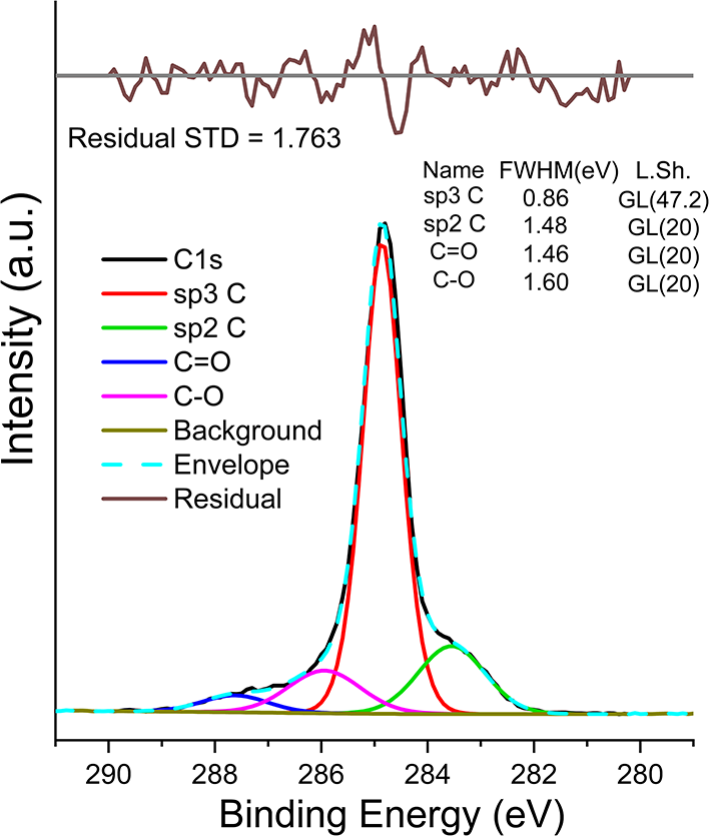
Results of peak-fitting C 1s spectra at a sample tilt angle of 10° for an O-terminated diamond.

**Figure 3:**
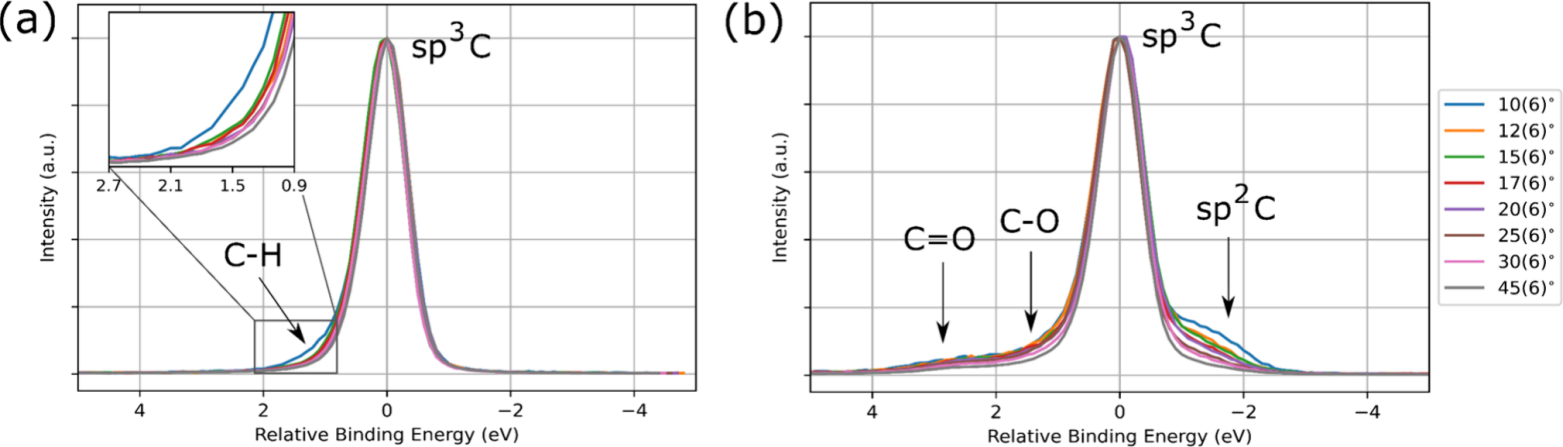
(a) C 1s spectra for H-terminated diamond at different sample tilt angles. (b) C 1s spectra for O-terminated diamond at different sample tilt angles.

For the purpose of a meaningful comparison of both diamonds across varying sample tilt angles, data are shown in terms of the relative binding energy (RBE), with all spectra shown relative to the bulk sp^3^ C peak. The values of the RBE for the two different surface treatments are provided in Table 1, with a discussion regarding the origin of the peak shifting reserved in the Supporting Information (Section S.4).

**Table 1:** Relative Binding Energy Values for All Chemical Species Identified in Both H- and O-Terminated Diamond Samples

H-terminated diamond	
Chemical species	Relative binding energy (eV)
sp^3^ C	0.00
C–H	0.46

O-terminated diamond	
Chemical species	Relative binding energy (eV)
sp^3^ C	0.00
sp^2^ C	–1.24 ± 0.05
C–O	1.09 ± 0.09
C=O	2.65 ± 0.07

O-terminated diamond (Figure 2b) displays more variations in surface chemistry when compared to the chemical homogeneity of the H-terminated diamond. Consistent with prior works on O-termination on SCD, we observe the presence of four chemical groups, sp^2^ C, sp^3^ C, and two oxygen functional groups denoted as C–O and C=O.^46,38,53,32^ The singly bonded C–O peak comprises the ether and hydroxyl bonds on the diamond surface, while the C=O peak is indicative of carbonyl bonding.

Both sets of spectra also change differently across varying sample tilt angles. The zoomed-in plot of the H-terminated data in Figure 2a shows the sudden change in the C–H peak region between the spectra taken at 10(6)° and the rest of the angles, with the data taken at 12(6)° and onward showing a gradual decline in the C–H peak. Given the proximity of the C–H peak position to the sp^3^ C peak at angles above 12°, the only peak fitting that was performed for the H-terminated data was at 10°, as attempting to perform peak fitting for any angles above 12° was nontrivial due to the ambiguity in peak position between sp^3^ C and C–H. We ascertain that at a sample tilt angle of 10(6)°, corresponding to 99% photoelectron escape probability at a depth of 3.0 pm, the sample exhibits a surface predominantly terminated by H. However, as the angle changes to 12(6)°, the 99% photoelectron escape probability depth is now 3.6 pm, and although the C–H portion is still present, the contribution is noticeably smaller when compared to the data at 10(6)°. This stark change in the C 1s spectra suggests that the H-termination is constrained to the diamond surface on the order of an atomic layer. By comparison at similar photoelectron escape depths, the O-terminated data show a peak shape that exhibits more gradual changes as the spectra begin to penetrate deeper into the diamond. Based on these findings, we confirm prior literature that has ascertained that an H-terminated diamond surface results in a chemically homogenized environment when compared to its O-terminated counterpart.^54^

### Angle-Dependent CKLL Spectra and D-Parameter Analysis

The acquisition and analysis of the CKLL spectra have been useful in the carbon community to determine the sp^2^ C content of materials through the calculation of the D-parameter, a value which represents the amount of π electrons inherent in sp^2^ C^55^, and manifests as the difference between the minimum and maximum points in the kinetic energies of the first derivative of the CKLL spectra. As Lascovich et al. showed through comparison of graphite, diamond, and amorphous carbon samples, the D-parameter tends toward values of approximately 22.5 eV for graphite, while for diamond, this will decrease to roughly 14.0 eV.^55–57^ In essence, sp^2^ C-rich materials will tend toward higher D-parameter values, while materials heavy in sp^3^ C will exhibit a lower D-parameter. Using this, it is possible to calculate the sp^2^/sp^3^ C fraction in a material via linear interpolation of the D-parameter from a range of about 14.0 to 22.5 eV.^56^ Although CKLL analysis has been performed on diamond-like materials, interpreting the sp^2^ C content on bulk diamond samples with the D-parameter comes with certain challenges. As evidenced in Figure 1, the inelastic mean free path (IMFP) of diamond for energies relevant to the C 1s and CKLL spectra are 1.71 and 0.490 nm, respectively. This means that for the same measurement, and assuming a constant sample tilt angle, a direct comparison between the sp^2^/sp^3^ C fraction that was derived from the C 1s peak fitting with that of the sp^2^/sp^3^ C value calculated from the D-parameter would not be possible due to a mismatch in the depth information between both spectra. In fact, this is a limitation of the CKLL analysis technique that has been observed previously in microcrystalline diamond films.^58^

However, given the surface sensitivity of the CKLL photoelectrons, one can probe the shallowest depths in SCD in a way that is not possible with analysis of the C 1s spectra. One of the most relevant pieces of information that can be extracted from this is the presence of a native sp^2^ C layer on the surface of the SCD.^35,38^ The data on an estimated thickness of this layer is sparse, but an approximate value based on experimental ARXPS data was calculated by assuming a three-layered model of the SCD surface consisting of a first layer comprised of C bonded to O in a 1:1 ratio, a second layer formed of solely sp^2^ C, and finally the bulk sp^3^ C diamond.^29^ The nondiamond region of the SCD surface, the first and second layer, were calculated to be 0.089 and 0.27 nm, respectively, making the cumulative amount of the non sp^3^ C region 0.36 nm.

Here, we arrive at a value of the thickness of the non sp^3^ C region without any assumptions of the native sp^2^ C depth or the C:O ratio on the surface. We do this by employing D-parameter analysis to track the evolution of the sp^2^/sp^3^ content as a function of sample tilt angle and by analyzing the ratio of the integrated peak areas. Shown in Figure 4a are the plots for the first derivative of the smoothened CKLL raw spectra taken for the O-terminated SCD sample at all sample tilt angles. All raw spectra were first smoothened with a modified form of the Whittaker smoothening method, known as the Whittaker–Eilers smoother,^44,45^ and then differentiated. The red points signify the highest and lowest points in the differentiated spectra, whose difference represents the D-parameter (details for select angles are shown in Figure 4c,d).

**Figure 4:**
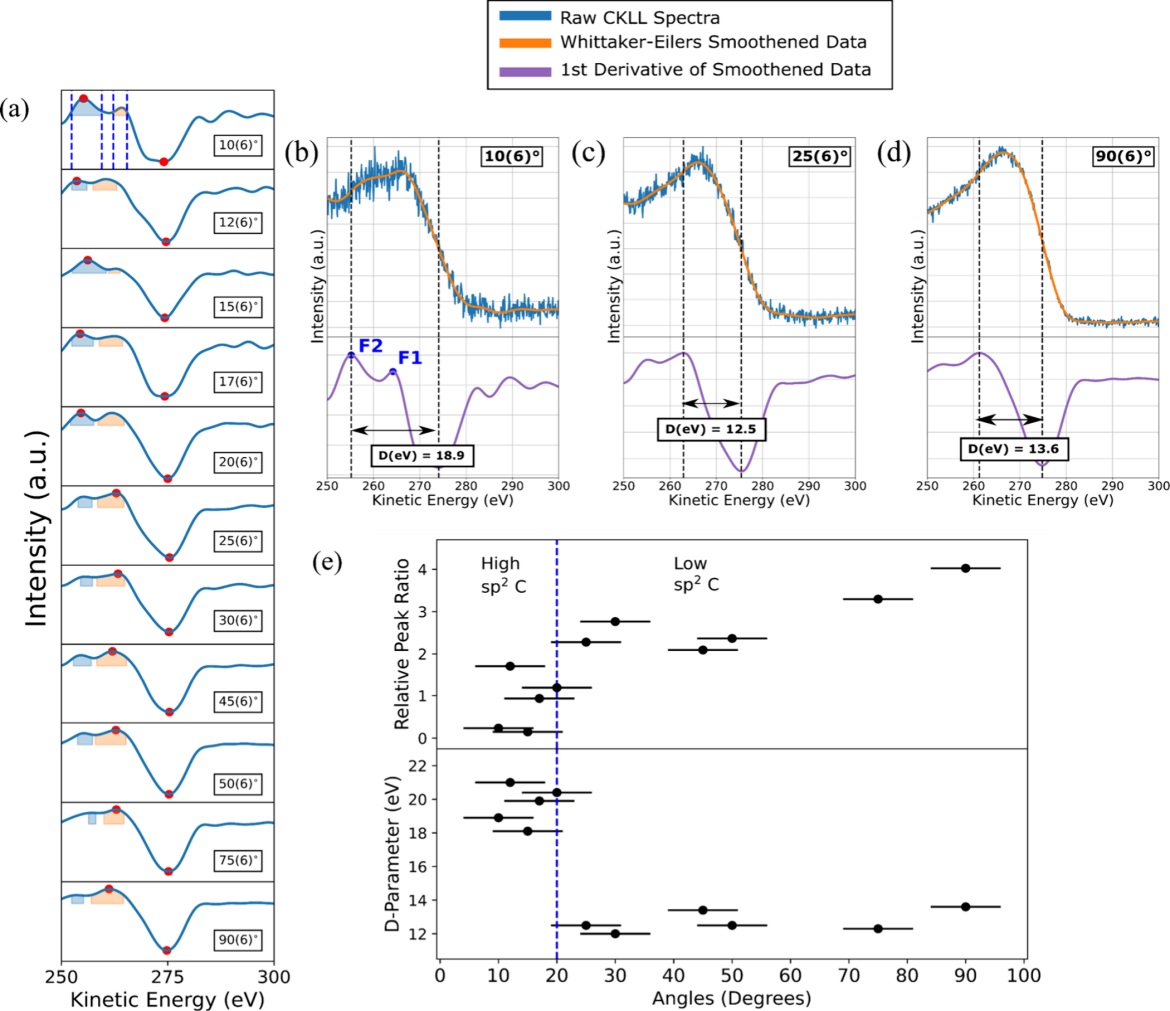
(a) First derivative of smoothened CKLL spectra of an O-terminated diamond for all sample-title angles. The data at 10(6)° shows an example of the peak finding algorithm that was employed for all of the smoothened data. For each subsequent angle, the shaded regions represent the integrated areas used to determine the relative peak ratio. (b) Raw CKLL spectra and first derivative of smoothened data shown with the calculated D-parameter at 10(6)°, corresponding to a photoelectron escape depth of 1.80 pm at an escape probability of 99% (c) Raw CKLL spectra and first derivative of smoothened data shown with the calculated D-parameter at 25(6)° corresponding to a photoelectron escape depth of 4.38 pm at an escape probability of 99%. (d) Raw CKLL spectra and first derivative of smoothened data shown with the calculated D-parameter at 90(6)° corresponding to a photoelectron escape depth of 6.59 pm at 99% escape probability. (e) (Top) Relative peak ratio and (bottom) calculated D-parameter values plotted as a function of sample tilt angles. Also shown are the regimes of high sp^2^ and low sp^2^ C for which the transition angle is at 20(6)°.

In the differentiated spectra shown in Figure 4a, the shallowest angle, at 10(6)°, has two peaks at around 255 (F2) and 263 eV (F1). These peaks are present at energies below 270 eV in the CKLL spectra and have been observed in prior works with high sp^2^ C materials, such as graphite samples^59,58^ and amorphous C.^40^ Prior work has attributed the F2 and F1 energy peaks to representing the σ–σ and σ–π partial local electron densities, respectively.^60^ The prominence of the F2 feature at angles at or below 20° suggests that the shallowest portion of the SCD is comprised of a high amount of π bonds that are common in sp^2^ C. To the best of our knowledge, correlation between the CKLL line shape and the density of states of the diamond orbitals, similar to what has been accomplished for the graphite CKLL spectra, is not well understood,^61^ but differentiated Auger spectra in diamond tend to show the higher-energy peak becoming more dominant.^61,58,59^ As the sample tilt angle increases, we observe the relative peak intensities change between the two peaks, with F2 being prominent at shallow angles and the high-energy peak becoming more prominent for deeper angles (indicating that the probed regions are increasingly sp^3^-like). By monitoring the change in the ratio of the two integrated peak area intensities (shaded regions shown in Figure 4a) as a function of sample tilt angle, we have devised a complementary technique to D-parameter analysis to monitor the change between the sp^2^ and sp^3^ regions present on the diamond surface.

These peak area ratios and D-parameter values for all angles are then plotted in Figure 4e, from which we can see that the relative peak ratio (upper plot) gradually increases and eventually plateaus as the sample tilt angle increases. The knee of this plateauing curve happens at roughly 20(6)°, which coincides with the transition from the high to low D values (lower plot). The higher D value region represents the depth in the SCD layer with a high sp^2^ C content, which is predominant at 10(6)° to 20(6)°. At 25(6)°, this value drops immediately to 12.5 eV and then is roughly constant at 13.0 eV for all subsequent angles.

In conclusion, the CKLL data on the O-terminated diamond are indicative of different sp^2^/sp^3^ C regions on the diamond for which representative raw CKLL spectra and the smoothened first derivative data are illustrated in Figure 4b–d. Labeled on the subfigures are the maximum and minimum values in the differentiated spectra, shown as vertical dotted lines, and the D-parameter values, shown as the horizontal arrows between the dotted lines and labeled accordingly underneath each of the arrows. Three angles are shown in the subfigures, being 10(6), 25(6), and 90(6)°. Each of these angles represents different regions of carbon in the SCD layering. First, an amorphous C region rich in sp^2^ C bonds, second, the point at which the materials start to shift from being composed of mostly sp^2^ C to sp^3^ C, and finally, the bulk diamond, predominantly composed of sp^3^ C bonds. From this, we can discern that the amorphous sp^2^ C layer extends within roughly 10(6) to 20(6)°, otherwise labeled as the high-sp^2^C region in Figure 4e. Past 20(6)°, our D values start to drop and are centered around 13.0 eV. The observation that there is a sudden drop in D-parameter values corroborates prior ARXPS work that deduced a model of the O-terminated SCD surface composed of a shallow region of sp^2^ amorphous carbon followed by the bulk sp^3^ diamond region.^29^ This region is labeled as the low-sp^2^C region, composed of angles between 25(6) and 90(6)°. By comparison, H-terminated diamond surfaces exhibit this transition at 10(6)°, suggesting that any sp^2^ C present is confined to the shallowest portion of the material (see the Supporting Information, Section S.6, for CKLL data and subsequent analysis of H-terminated samples). In combination with the lack of oxygen functional groups found on the XPS C 1s narrow-scan spectra for H-terminated diamond, we attribute the source sp^2^C to ubiquitous organic carbon contamination, also known as adventitious carbon.

### Calculation of the sp^2^ C Layer

With this information, we can now estimate an upper bound of the depth of the amorphous carbon sp^2^ C region on the SCD sample. We assume here that the D-parameter that is measured from our sample follows the same linear relation between D values and % sp^2^ C content for other carbon allotropes.^59^ Using this relation, we can relate the amount of sp^2^ C to the photon electron escape probability using eq 2 (see the Supporting Information, Section S.8, for derivation).

$$\%{\mathrm{sp}}^{2}C=\frac{\int_{0}^{T}e^{-z/\left(\lambda_{a-C}\cdot \sin(\theta )\right)}dz}{\int_{0}^{T}e^{-z/\left(\lambda_{a-C}\cdot \sin(\theta )\right)}dz+\int_{0}^{\infty }e^{-T/\left(\lambda_{a-C}\cdot \sin(\theta )\right)}\times e^{-z/\left(\lambda_{{\mathrm{sp}}^{3}C}\cdot \sin(\theta )\right)}dz}$$ 2

The left-hand of the equation, % sp^2^ C is the percentage of the sp^2^ C content, obtained from the D-parameter.^59^ On the right-hand side of the equation shows a ratio of the integrated photoelectron escape probabilities. The numerator relates only to the integrated photoelectron escape probability from the sp^2^ C layer, while the denominator is the integrated photoelectron escape probability from both the bulk diamond and sp^2^ C layer.

The T value in the integration bounds represents the thickness of the amorphous sp^2^ C layer, the λ_a–C_ value is the IMFP for amorphous carbon, and λ_sp^3^C_ is the IMFP for diamond. Note that the denominator is the total sum of the integrated photoelectron escape probability from the sp^2^ C layer and the bulk diamond, with the integral from zero to infinity representing the total probability of a photoelectron exiting both the bulk diamond and the sp^2^ C layer. The $e^{-T/\left(\lambda_{a-C}\cdot \sin(\theta )\right)}$ expression is equal to the photoelectron escape probability right at the sp^2^ C layer, which is then multiplied by $e^{-z/\left(\lambda_{sp^{3}C}\cdot \sin(\theta )\right)}$ to yield the probability of a photoelectron exiting the diamond and amorphous C layer. Solving this equation for T then results in the following

$$T(\theta ,\%{\mathrm{sp}}^{2}C)=-\lambda_{a-C}\cdot \sin(\theta )\ln\left(\frac{\lambda_{a-C}(\%{\mathrm{sp}}^{2}C-1)}{\lambda_{a-C}(\%{\mathrm{sp}}^{2}C-1)-\%{\mathrm{sp}}^{2}C\cdot \lambda_{{\mathrm{sp}}^{3}C}}\right)$$ 3

The amorphous sp^2^ C layer is then a function of the percentage of the sp^2^ C content and the sample tilt angle. Here, we assume that the values for both λ_a–C_ and λ_sp^3^C_, at values of 1.03 ± 0.11 and 0.490 ± 0.015 nm, respectively, are at a constant kinetic energy of 263.0 eV. Given a value of 20° (and an angle uncertainty of 6°), a D-parameter of 20.4 eV, corresponding to 74% of sp^2^ C, we attain a sp^2^ C layer depth of 0.3 ± 0.1 nm.

The implications of a model of the single-crystal diamond surface that consists first of an amorphized C layer means that the O functional groups are bonded to the sp^2^ C and not directly to the sp^3^ C. This is also evidenced by analyzing the relationships between the sp^2^/sp^3^ C fractions, the atomic percentage of the atomic fraction of the atomic percentage of the atomic percentage of the atomic area of the total O-functionalization area from the survey, and C 1s narrow-scan spectra. Figure 5a illustrates the atomic percentage of the O 1s plotted against the fractional amounts of sp^2^/sp^3^ C, where each data point has been color-coded with respect to the color map shown on the right-hand side of the subplot to signify the change in sample tilt angle that each point corresponds to. The data show that at the shallower angles the O 1s atomic percentage plateaus with respect to the amount of sp^2^ C in the sample. As the XPS probes deeper into the sample, the O 1s atomic percentage diminishes as the diamond becomes more sp^3^ C-rich. These results are consistent with the total O content in the diamond being constrained mostly to the sp^2^ C layer, and not necessarily the sp^3^ C portion.

**Figure 5:**
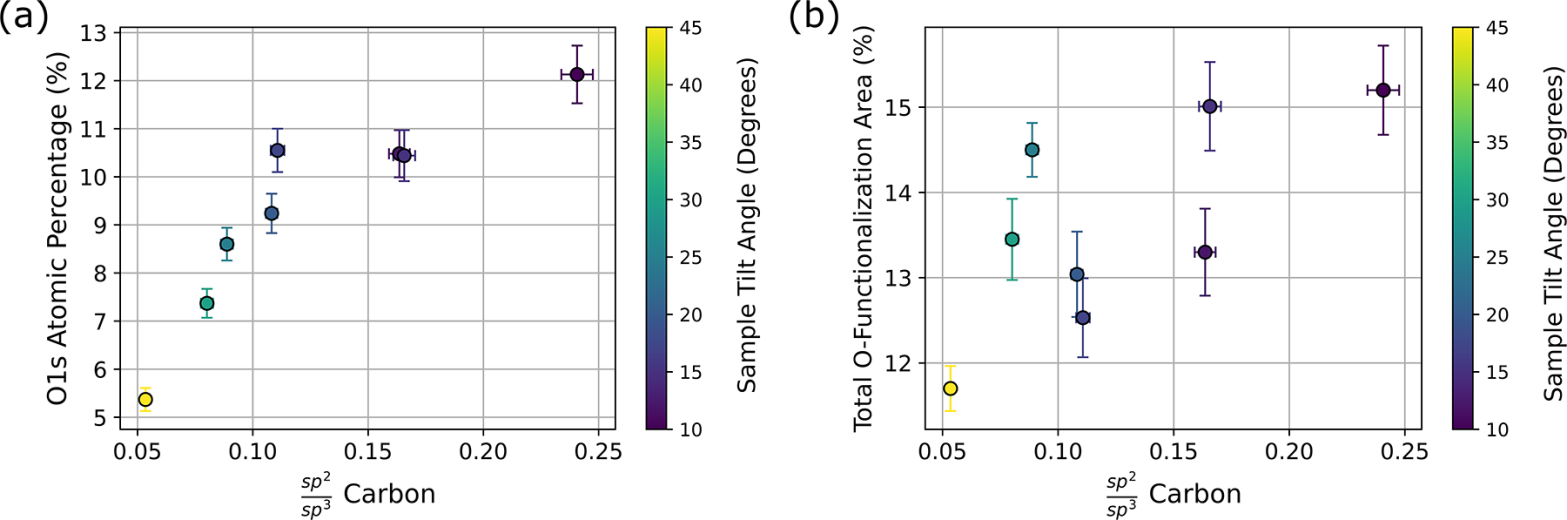
(a) sp^2^/sp^3^ C fraction shown alongside O 1s atomic percentage. (b) Total O-functionalization area shown as a function of fractional amounts of sp^2^/sp^3^ C. The color bar on the right-hand side of the plot represents the change in sample tilt angle for all data points on the figure.

Figure 5b shows the fractional amount of sp^2^/sp^3^ C against the total O-functionalization area percentage, with each data point color-coded to represent the corresponding sample tilt angle. At the shallower sample tilt angles, we see a sharp increase in the amount of total O-functionalization area percentage, which corresponds to increasing amounts of sp^2^ C. Similar to how the O 1s atomic percentage drops off in Figure 5a, we see a sharp decrease in O-functionalization as the sample transitions toward being sp^3^ C-rich. This means that the vast majority of the O functional groups are present on the sp^2^ C portions of the diamond. Taken together, Figure 5a,b illustrates that the O-rich portions of the diamond tend to be constrained on the sp^2^ C-rich regions. The reader will note that values of O 1s atomic percentage or total O-functionalization area percentage never fall down exactly to zero as the sp^2^ C decreases. This is due to the fact that, despite probing deeper into the diamond, some nonzero amount of photoelectrons still emanate from the superficial amorphous C layer, although most of them will correspond to the sp^3^ C-rich regions.

## Conclusions

This work represents a thorough study of the evolution of both the C 1s and CKLL spectra with ARXPS at varying sample tilt angles at 10(6) to 90(6)°. Analysis of the C 1s spectra for an O-terminated sample reveals higher amounts of sp^2^ C and functional groups present at the shallower angles that gradually wane as the photoelectrons begin exiting from the bulk diamond. The data for the C 1s spectra of the H-terminated sample reveal only the presence of sp^3^ C and C–H bonds with no sp^2^ C or functional groups, although the CKLL data for the H-terminated diamond do show evidence of a shallow layer of adventitious carbon, constrained to within 10° of the sample tilt angle. The lack of sp^2^ C on the C 1s spectra of the H-terminated sample is due to how the plasma H-termination process both performs some etching of the diamond and passivates the remaining dangling carbon bonds found on the surface, resulting in a stable layer of C–H bonds.

Finally, our analysis of the CKLL spectra reveals layering of different carbon allotropes, amorphous sp^2^ C and sp^3^ diamond C, through the calculation of the D-parameter at all sample tilt angles. By using the linear relation between the D-parameter and the percentage of the sp^2^ C content, and the integrated photoelectron escape probabilities through both the sp^2^ C and bulk diamond region, we calculate an amorphous sp^2^ C depth of 0.3 ± 0.1 nm. Compared to a prior ARXPS study by Alba et. al, which deduced a cumulative sp^2^ C and oxygen contribution of 0.36 nm on SCD based on assumptions of diamond surface layering, our value was obtained without relying on any prior assumptions.^29^ This represents only an 18% difference from their result. This likely means that for O-terminated SCD, the O functional groups are bonded not directly to the sp^3^ C diamond, but rather, they are bonded to the amorphous sp^2^ C layer. By utilizing the results from the survey scan and the peak fitting from the C 1s spectra, we observe that the total O content and the O-functionalization area percentage are constrained to the sp^2^ C-rich portions of the sample. Together, these results signify that the O is bonded directly to the sp^2^ C layer, and not directly to the sp^3^ C region. While this work utilizes aqueous mineral acids for oxygen termination, other oxygen termination approaches (e.g., involving oxygen exposure at elevated temperatures^34^) have also been reported nonzero amount of sp^2^ C, and thus we believe our conclusions can be extended to other O-terminated diamond surfaces.

Future work can focus on additional characterization experiments to probe the identity of the amorphous carbon layer. For example, a worthwhile study would be to compare the sp^2^C calculated from the D-parameter and compare it to the value attained from a C 1s peak fit. Although, such an experiment would require exceedingly accurate angular resolution to resolve minute differences in the change of both the CKLL and C 1s spectra across shallow angles on the diamond surface. This work would also benefit from depth-profiling experiments with the Near Edge X-ray Absorption Fine Structure (NEXAFS), in which one would have to use the Partial Electron Yield (PEY) mode to filter out non-Auger carbon electrons. However, this would require calculating the escape depths of the electrons in diamond at the particular voltage bias that is being applied in PEY mode,^62,63^ in order to gauge an accurate depth assessment. High-resolution electron energy loss spectroscopy (HREELS) could also be performed to probe shallow surface information on diamond, but these measurements should be performed alongside a Monte Carlo simulation of electron interactions on the diamond surface to ascertain the depth information from the spectra. Another issue that remains concerns the origin of the sp^2^ C layer in O-terminated diamond, which may benefit from computational modeling and experimental work that tracks the evolution of this sp^2^ C layer formation by oxidation of an orderly H-terminated diamond. For example, prior scanning transmission electron microscope (STEM) studies tracked the evolution of how the shallowest layer of C on laser-ablated SCD changed with respect to thermal oxidation in air and wet chemistry treatments.^64^ A prior molecular dynamics (MD) simulation showed that, given an orderly sp^3^ C diamond surface, exposure to mechanical polishing results in the accumulation of an amorphous layer predominantly composed of sp^2^ and sp^3^ C amorphous phases.^65^ A similar study that analyzes how an sp^3^ C diamond surface would be altered following exposure to an oxidation treatment would also be helpful in examining the origin of this sp^2^ C layer.

## Supplementary Material



## Notes

AFM measurements and additional XPS results and analysis on all diamond surfaces studied in this work, as well as details of the sp^2^ C layer thickness (PDF)
