# Supplementary material for: Subnanometer Thick Native sp2 Carbon on Oxidized Diamond Surfaces
Source: Langmuir. 2025 Oct 1;41(40):27133–42. doi: 10.1021/acs.langmuir.5c02616 (PMC12530048; doi:10.1021/acs.langmuir.5c02616)
Supplement: Supplementary file 1 [file la5c02616_si_001.pdf]

Sub-nanometer thick native sp^2 carbon on oxidized diamond surfaces

Ricardo Vidrio[1†], Cesar Saucedo[2], Vincenzo Lordi[3], Shimon Kolkowitz[4], Keith G. Ray[3], Robert J. Hamers[2], Jennifer T. Choy[1*]

[1]1415 Engineering Dr, Madison WI 53706, Department of Electrical and Computer Engineering, University of Wisconsin-Madison

[2]1101 University Ave, Madison WI 53706, Department of Chemistry, University of Wisconsin-Madison

[3]7000 East Ave, Livermore CA 94550, Lawrence Livermore National Laboratory

[4]Physics South Hall, Berkeley, CA 94720-7300, Department of Physics, University of California, Berkeley

Table of Contents

S.1 AFM data on tri-acid cleaned samples and hydrogen-terminated samples.......................2

S.2 Calculation of photoelectron refraction effects 3

S.3 C1s deconvolution results 4

S.4 Peak shifting effects 4

S.5 Assigning maximum and minimum values in CKLL spectra 5

S.6 Comparison between H and O terminated (100) single crystal diamond data 7

S.7 Identifying peak regions for CKLL spectra 9

S.8 Equation that calculates sp^2 C layer thickness 11

S.9 Comparison of H and O terminated samples with as-received diamond 12

[†]vidrio@wisc.edu
[**]jennifer.choy@wisc.edu

Supplementary

S.1 AFM data on tri-acid cleaned samples and hydrogen-terminated samples

Figure S1 shows two representative images of AFM performed on an oxygen-terminated diamond sample treated with the tri-acid cleaned method. A Bruker Icon AFM was used to characterize the surface roughness and topology of diamond samples. The Ra value for the leftmost figure is 0.210 nm and the Ra value for the rightmost figure is 0.277 nm. Furthermore, Figure S2 shows comparisons between tri-acid cleaned diamond and H-terminated diamond, which exhibit roughness values of less than 1 nm.

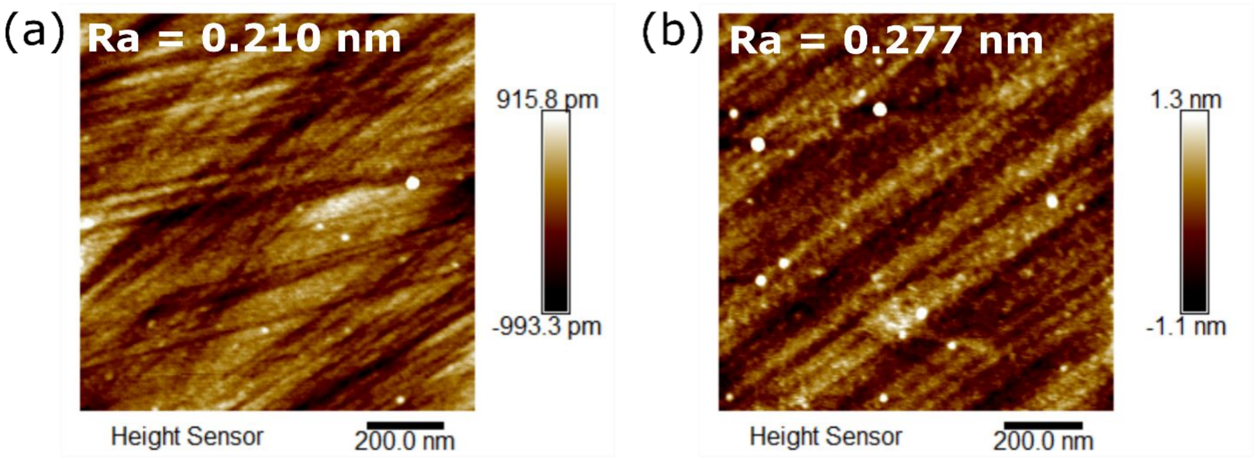

Figure S1: Two representative AFM images from two different diamond samples oxygen-terminated via the tri-acid cleaned method. (a) AFM image of oxygen-terminated diamond with a surface roughness of Ra = 0.210 nm (b) AFM image of oxygen-terminated with identical treatment and an Ra value of 0.277 nm

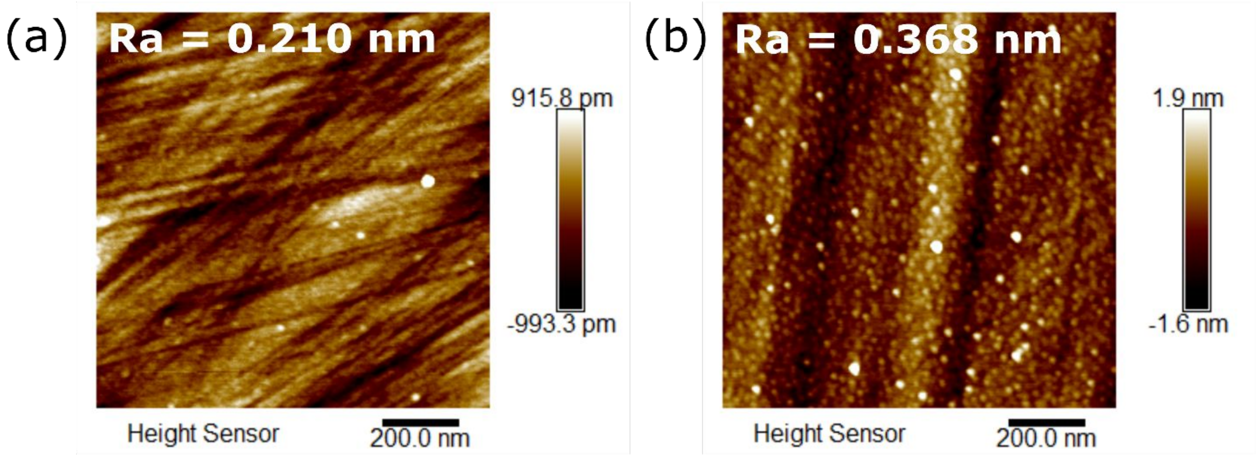

Figure S2: Two representative AFM images from two different diamond samples. (a) tri-acid cleaned O-terminated (b) H-terminated diamond.

S.2 Calculation of photoelectron refraction effects

Given a sample that is exposed to the vacuum in the XPS chamber the following equation applies between the photoelectron take off angle and the inner incidence angle (Cazaux, J. Electron Spectrosc, 187, 2013).

$$\sqrt{E_s}\cos(\theta') = \sqrt{E_k}\cos(\theta)$$

$$E_s = E_k + \chi$$

where here E_s is the effective electron kinetic energy which is equal to the electron kinetic energy E_k plus the surface electron affinity χ. θ' is the inner incidence angle in the sample and θ is the take off angle for the photoelectrons in the vacuum. Electron affinity values for tri-acid cleaned (100) diamond surfaces have been taken from UPS measurements performed by Sangwatesin et. al (2019). Given that UPS spectroscopy is surface sensitive to roughly the first 1 – 5 nm, we feel that this value is appropriate to use for our ARXPS data. The χ is taken to be as 0.92 eV. Inputting this into the prior equation at electron kinetic energies of 1202 eV (for C1s spectra) and 260 eV (for CKLL spectra) we find that the take-off angle only deviates from the sample tilt angle by less than 1˚. Given that the angle uncertainty from the exclusion of the optical aperture is 6˚, the error from the absence is greater than the error from the electron refraction effect, which is why it was excluded from the calculation of the amorphous sp^2 C layer.

S.3 C1s deconvolution results

Figure S3 shows the results of the fitting at sample tilt angles of 12, 20, and 45˚. The methodology is described in Section 2.4 in the main text.

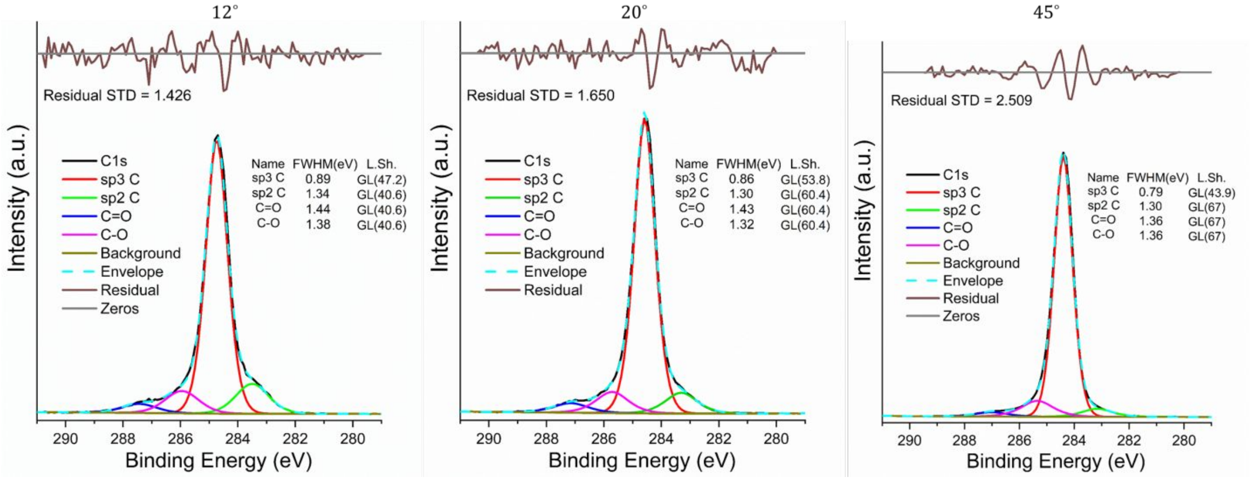

Figure S3: Fit results for C1s spectra on O-terminated diamond at various tilt angles.

S.4 Peak shifting effects

Peak-shifting effects were visible in the carbon photoelectron spectra for both H and O terminated diamond samples despite the flood gun being utilized. The peak-shifting has been seen in prior work for H-terminated diamond, as the C-H bonds contribute to upward band bending, which in turn reduces the barrier needed for electron emission from the sp^3 C peak position , but becomes particularly more noticeable here due to how the band structure changes as the photoelectrons start out emanating from the rich C-H layer at the lower angles and then transition towards the bulk sp^3 C at higher angles [1]. We record the maximum peak shifted across the H-terminated diamond sample measurements as 0.40 eV.

The origin of the peak shifts in the oxygen terminated sample most likely results from the effects of vertical differential charging (VDC) which stems from the layered chemical structure of the diamond sample, which features a prominent presence of electrically conductive sp^2 C in the shallower regions at lower angles, followed by the insulating sp^3 C in the bulk at higher angles. VDC effects have been observed in heterogenous materials that feature an insulating thin overlayer followed by a bulk conducting substrate, where in those works, peak shifting was noticeable even with the implementation of charge neutralization methods [2] [3] [4]. Here we see the same shifting behavior, albeit conversely with a thin electrically conductive layer followed by a bulk insulator.

At the lower angles the material behaves like an electrical conductor, and therefore the charge neutralization effects of the flood gun would overcompensate for any surface charging effects, resulting in lower BE values for the bulk sp^3 C peak. Contrarily, as the x-rays penetrate deeper into the diamond, and the photoelectrons originate from the bulk sp^3 C region, the kinetic energies of the exiting photoelectrons will be slightly hindered as they depart from an insulating material, resulting in higher BE values. The maximum value in the peak shifts for the O-terminated sample was recorded as 0.30 eV.

S.5 Assigning maximum and minimum values in CKLL spectra

The reader will note that in Figure S4 at 12(6)˚ there are both a green and red point present at lower kinetic energies. The green point represents the highest value that was chosen for the calculation of the D-parameter, as it was found that the maximum (the red point to the left of the green) yielded a D value of 14.2 eV, which in turn was inconsistent with the rest of the data between 10(6) to 20(6)˚. This represents some of the inherent limitations of the D-parameter analysis technique, as it is imperative that one smoothens their CKLL raw spectra before differentiating, as the auger spectra is normally too noisy to just differentiate outright. Improperly applying a smoothening method to the CKLL spectra can lead to erroneous D-parameter values, via either under or over smoothening. In this case, it was found that at 12(6)˚ the height difference between the green and red point was only about 0.16 a.u. apart. Seeing as how the D-parameter at 12(6)˚ was the only value between 10(6)˚ to 20(6)˚ to not be higher than 18 eV, it was determined that the green point was the true maximum value, with the other maximum value being present there only as an artifact of the smoothening method. After factoring in this change, the revised D-parameter was calculated to be 21.0 eV.

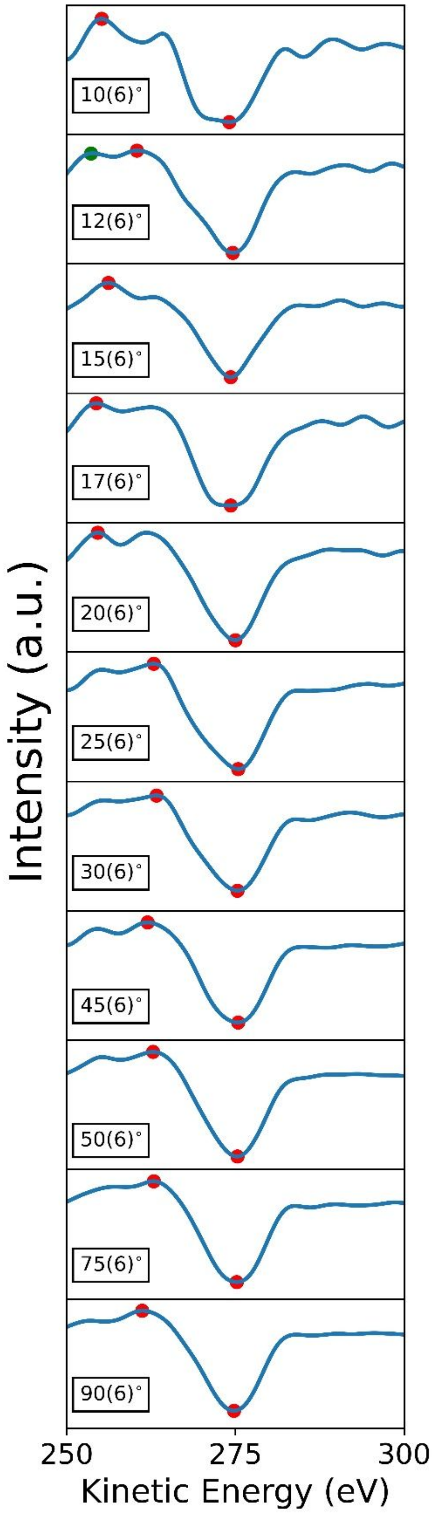

Figure S4: Smoothened and differentiated CKLL spectra for all sample tilt angles for O-terminated diamond. The data at 12(6)˚ show two points, one green and the other red. The red point illustrates the maxima of the spectra, whereas the green point is the second highest maxima. Given the difference between both points, the green point was assigned as the true maxima and is present in the main text of this work.

S.6 Comparison between H and O terminated (100) single crystal diamond data

Figure S5 shows the smoothened and differentiated CKLL spectra data comparisons between H and O terminated diamond at various sample tilt angles. XPS experimental parameters were similar, albeit data was only collected from 246.6 eV to 286.6 eV for the H-terminated samples, whereas data was collected from 246.6 eV to 311.6 eV for the O-terminated samples. D-parameter analysis was applied on both data sets. The results of the analysis reveal a shallow most layer of sp^2 carbon on the H-terminated samples roughly 0.1 nm in depth. Given that the H-terminated XPS survey spectra showed zero trace of oxygen, the source of this high sp^2 C rich signal is most likely adventitious carbon. This is also shown graphically on Figure S6, as the sample tilt angle at which the D-parameter drops to a lower value, of roughly 13 eV, happens at 12 degrees, a shallower angle when compared to the O-terminated data. In this case, once Auger electrons begin to emanate from the C-H surface termination, as opposed to the adventitious carbon layer, we see that the D-parameter has transitioned to values that are indicative of sp^3 bonding. As the sample tilt angle increases, and the CKLL signal emanates from the diamond rich region this value remains largely unchanged.

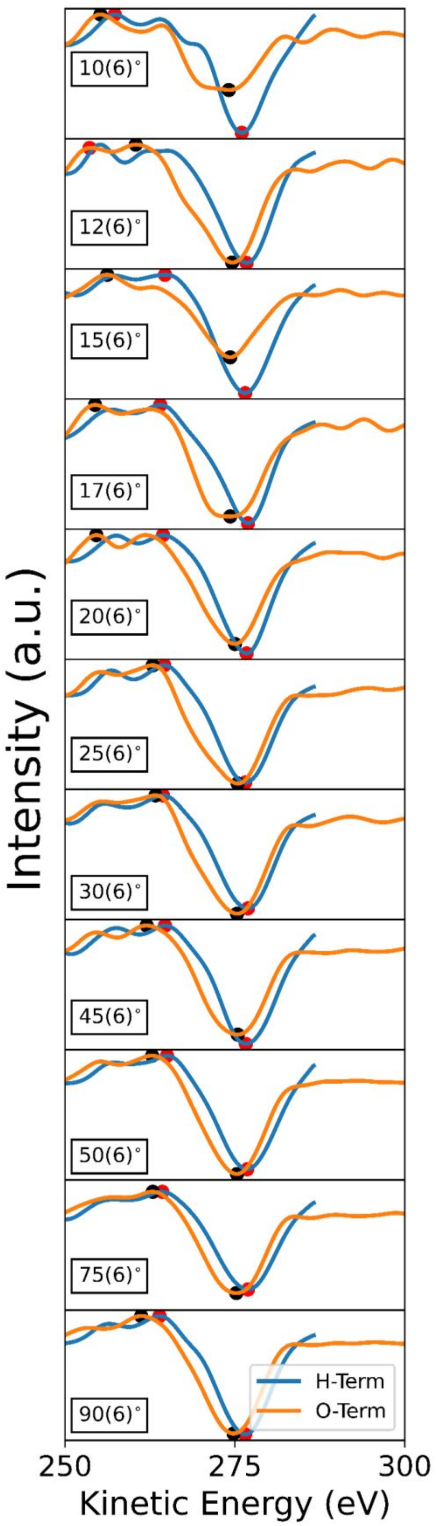

Figure S5: Smoothed and differentiated CKLL spectra for both H and O terminated diamond data. Point at 10(6)˚ shows both diamond samples with high amounts of sp^2 C. We attribute the origin of the sp^2 C present on the H-terminated diamond sample at 10(6)˚ to be due to adventitious carbon.

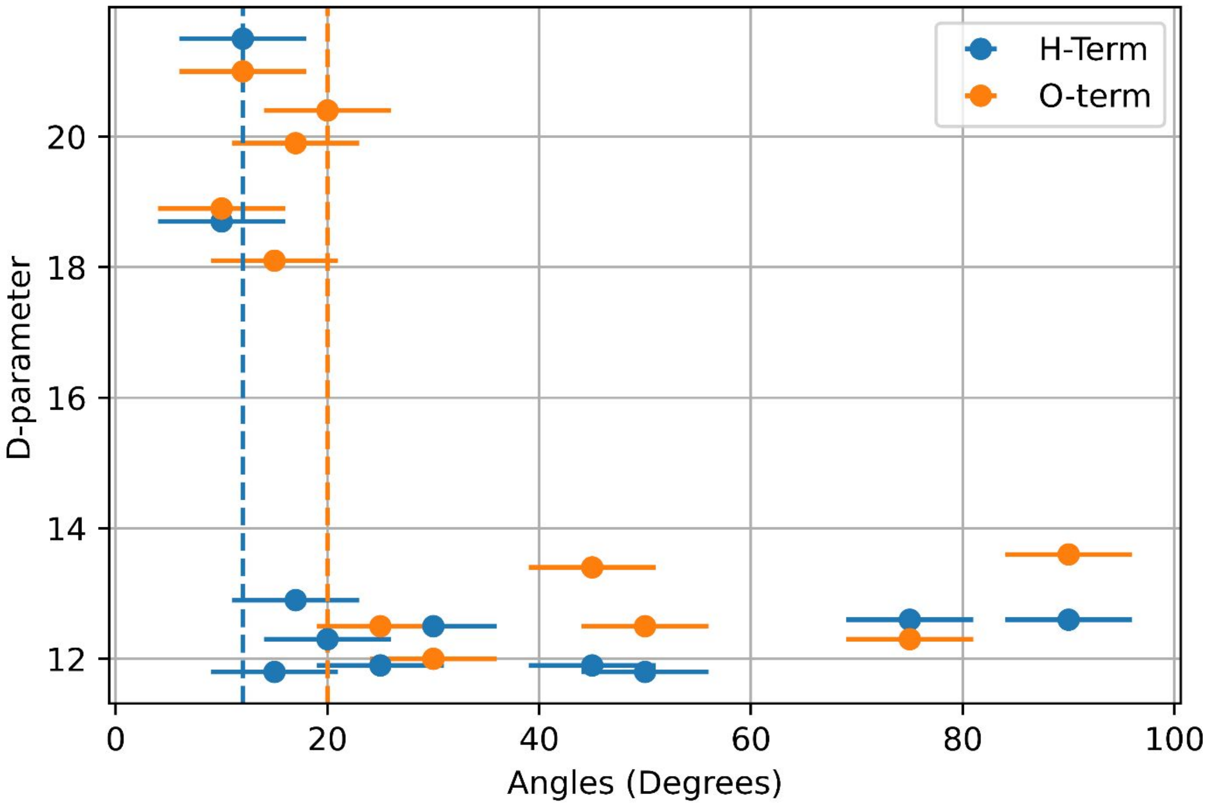

Figure S6: D-parameter values plotted against sample tilt angle for both H and O terminated diamond. Vertical dotted lines show the angles at which the sample transitions to rich sp^3 bonded regions.

S.7 Identifying peak regions for CKLL spectra

Figure S7 shows the results of the python script that identified the peak information of the processed CKLL spectra to calculate the ratio of the peak intensities to better understand the sp^2/sp^3 transition on the diamond surface.

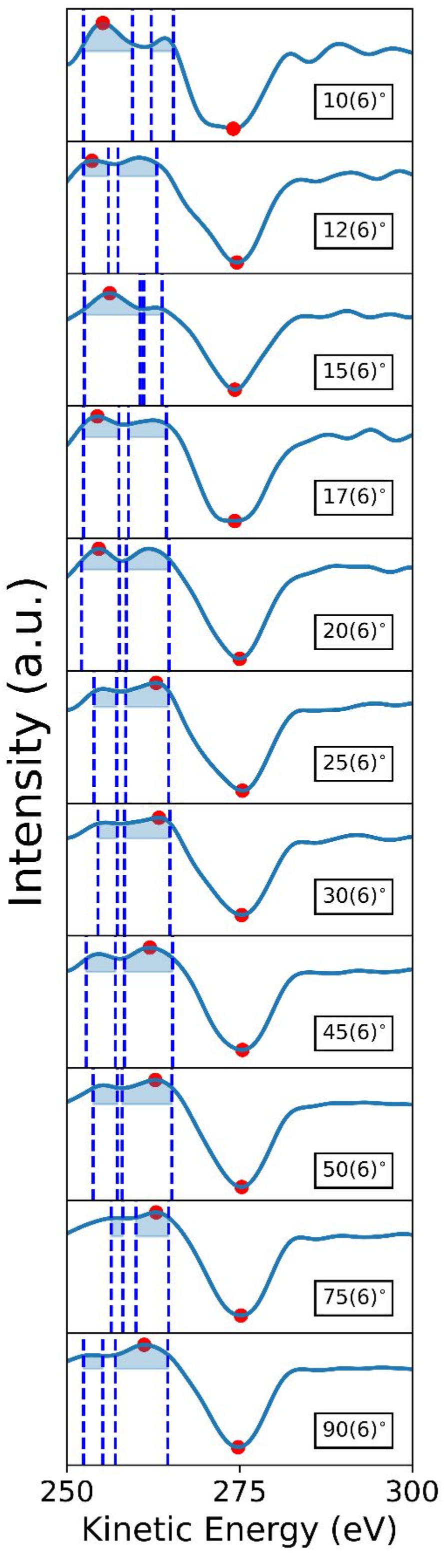

Figure S7: Processed CKLL spectra for all sample tilt angles. The dashed blue vertical lines are the integration bounds used for calculating the relative peak ratios. The blue shaded areas represent the region where the areas were integrated, and the corresponding ratios calculated.

S.8 Equation that calculates sp^2 C layer thickness

To get a value for the thickness of the amorphous the following equation (shown below) was used. Here $\%sp^2C$ is the percentage of sp^2 C calculated from the linear relation of the D-parameter [5], T is the thickness of the amorphous carbon layer, λ is the inelastic mean free path (IMFP) for both amorphous carbon (a-c) and diamond (sp^3C), θ is the sample tilt angle, and z is the photoelectron escape depth. The premise here is that the right hand side is equal to ratio of the integrated photoelectron escape probabilities, where the numerator represents the integration over the whole of the amorphous carbon layer (from 0 to T), and the denominator represents integrating over the entirety of the bulk diamond and the amorphous carbon layer. The denominator has two integrals, the first integral (with integration bounds from 0 to T) represents the integrated probability of escaping from the amorphous carbon layer, and the second portion of the integral represents the integrated photoelectron escape probability of escaping from the bulk diamond. To get the total probability of a photoelectron escaping from the amorphous carbon layer and the bulk diamond, these two probabilities are multiplied together with the first term $e^{\frac{-T}{\lambda_{a-c}\cdot\sin(\theta)}}$ represents an electron from the bulk diamond making it through the amorphous carbon layer, and the second term $e^{\frac{-z}{\lambda_{sp^3C}\cdot\sin(\theta)}}$ represents the probability of an electron escaping through the bulk diamond. This ratio is equivalent to the percentage of sp^2 C present on this amorphous carbon layer.

$$\%sp^2C = \frac{\int_0^T e^{\frac{-z}{\lambda_{a-c}\cdot\sin(\theta)}}dz}{\int_0^T e^{\frac{-z}{\lambda_{a-c}\cdot\sin(\theta)}}dz + \int_0^\infty e^{\frac{-T}{\lambda_{a-c}\cdot\sin(\theta)}} \cdot e^{\frac{-z}{\lambda_{sp^3C}\cdot\sin(\theta)}}dz}$$

This equation can then be solved for T. To do so, one will need to perform the necessary integrals and apply algebra to solve for T. The following equation is the result.

$$T(\theta, \%sp^2C) = -\lambda_{a-C}\cdot\sin(\theta)\ln\left(\frac{\lambda_{a-C}(\%sp^2C-1)}{\lambda_{a-C}(\%sp^2C-1)-\%sp^2C\cdot\lambda_{sp^3C}}\right)$$

The value for T can then be solved as all variables on the right hand side of the equation are known. Here it is pertinent to use electron kinetic energies of 263 eV, as all electron spectra analyzed were Auger electrons.

S.9 Comparison of H and O terminated samples with as-received diamond

Shown below are data for O and H terminated diamond samples compared against as-received diamond. All data are at constant sample tilt angles of 45˚. Present on this data is text specifying regions corresponding to sp^2 C, sp^3 C, singly bonded carbon C-O, C-H bonds, and doubly bonded carbon C= O. One will note that the as-receive diamond had noticeable amounts of sp^2 C, as well as a more functionalized surface when compared to both the O and H terminated diamonds. Given that the O1s atomic percentage for the as-received diamond was over 9%, we attribute this to sources of adventitious carbon from the packaging process. Furthermore, the sample has visible amounts of molecular contamination visible as Si 2p and 2s peaks, which only further increases the oxygen amount. In comparison the H-terminated surface had no visible oxygen and the O-terminated sample had 5.37%.

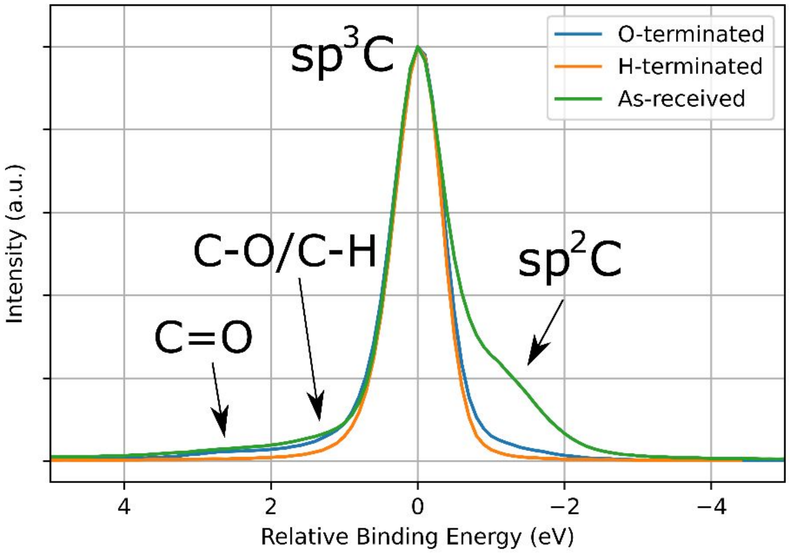

Figure S8: Comparison of C1s spectra for three different diamond samples, O-terminated, H-terminated, and as-received diamonds plotted with respect to their relative binding energies (RBE).
